# Pain Following the Use of Anesthesia Formulation Among Individuals Undergoing Cataract Surgery: A Randomized Controlled Trial

**DOI:** 10.3389/fphar.2020.00440

**Published:** 2020-04-16

**Authors:** Mario D. Toro, Dominika Nowakowska, Agnieszka Brzozowska, Michele Reibaldi, Teresio Avitabile, Claudio Bucolo, Paolo Murabito, Clara Chisari, Katarzyna Nowomiejska, Robert Rejdak

**Affiliations:** ^1^Department of General Ophthalmology, Medical University of Lublin, Lublin, Poland; ^2^Faculty of Medicine, Collegium Medicum, Cardinal Stefan Wyszynski University, Warsaw, Poland; ^3^Department of Mathematics and Medical Biostatistics, Medical University of Lublin, Lublin, Poland; ^4^Department of Ophthalmology, School of Medicine, University of Catania, Catania, Italy; ^5^Department of Biomedical and Biotechnological Sciences, School of Medicine, University of Catania, Catania, Italy; ^6^Department of General Surgery and Surgical Specialties, Division of Anesthesiology, Azienda Ospedaliero-Universitaria Policlinico, Catania, Italy; ^7^Department “GF. Ingrassia”, Section of Neurosciences, University of Catania, Catania, Italy

**Keywords:** cataract surgery, pain, ophthalmologic anesthesia, intraocular anesthesia, tropicamide, phenylephrine, lidocaine

## Abstract

**Purpose:**

To assess the pain intensity of two intracameral anesthetic solutions in patients undergoing cataract surgery and evaluate the factors influencing the patients’ postoperative activities.

**Methods:**

Sixty-two patients undergoing cataract surgery were randomized to receive the study drug – a manufactured solution of 0.02% tropicamide/0.31% phenylephrine/1% lidocaine (Mydrane) or a traditional anesthetic formulation - solution of 1% lidocaine/0.025% adrenaline as an intraocular anesthetic. The pain intensity was assessed by Visual Analog Scale for Pain (VAS Pain) and Brief Pain Inventory-short form (BPI) on the next day after the surgery.

**Results:**

The mean pain score measured preoperatively with VAS Pain was 0.34 in Mydrane group and 0.09 in the reference group (*p* = 0.51). There were no statistically significant differences between the two anesthetic methods with respect to pain intensity during the surgery (*p* = 0.94) and the influence of pain during the last 24 h on activity (*p* = 0.79), mood (*p* = 0.31), social contacts (*p* = 0.29), sleep (*p* = 0.5) and the joy of life (*p* = 0.39). Additionally, there was no statistically significant influence of age, sex, lateralization, co-existing ophthalmological diseases (*p* = 0.98) and post-operative complications (*p* = 0.4) on the experienced pain measured during the surgery and in the last 24 h.

**Conclusions:**

New commercially available intraocular anesthetic solution (Mydrane™) seems to be as effective as off-label traditional anesthetic formulation, in reducing the pain experienced during cataract surgery under topical anesthesia.

## Introduction

Since the 1990s, cataract surgery has progressed to the modern technique of phacoemulsification that involves a small corneal incision and the implantation of a foldable intraocular lens (IOL). This surgical procedure no longer requires complete akinesia, thus, encouraging the use of less invasive anesthetic modalities (Malik et al., 2010).

In the last decade, peribulbar, retrobulbar, and sub-Tenon’s anesthesia were the most popular techniques used during cataract surgery (Sanabria et al., 2013). However, some complications of these methods may be observed. Nowadays, different local anesthesia (topical and intracameral) are currently used. Eye drops based on esters such as oxybuprocaine or amethocaine, and amides such as lidocaine are the most common used topical agents for anesthesia of the cornea and conjunctiva (Malik et al., 2010). Intracameral anesthesia is often used as an adjunct to topical anesthesia to improve the effect of topical anesthesia alone. The most popular anesthetic injected into the anterior chamber is preservative-free lidocaine at different concentrations 0.5%, 0.75% or 1% (Sanabria et al., 2013). Lidocaine was discovered in 1946 and it is on the list of medications proposed by the World Health Organization to meet the most important needs in a basic healthcare system; it can be administered intravenously, subcutaneously, topically, and orally.^1^

The new NICE guideline (2017) for the management of cataracts in adults offers the possibility of use intracameral anesthesia for cataract surgery.^2^ Intracameral agents are often based on 1.5% phenylephrine alone or in combination with cyclopentolate 0.1% (Myers and Shugar, 2009; Cakmak et al., 2010; Tan et al., 2011; Hovanesian et al., 2015). So far, an off-label formulation based on 1% of lidocaine (anesthetic) and adrenaline 0.025% (mydriatic) has been used as golden standard regimen during cataract surgery.

The study drug - Mydrane™ (Laboratoires Théa, Clermont-Ferrand, France) is a fixed-dose mydriatic/anesthetic combination (0.02% tropicamide/0.31% phenylephrine/1% lidocaine) preservative-free, approved for intracameral use during cataract surgery. The study drug is a very appealing formulation because it represents a safe and effective combination of anesthetic and mydriatics (Labetoulle et al., 2016). Furthermore, there is a remarkable advantage in terms of costs (Davey et al., 2018).

Visual Analog Scale (VAS) for Pain and Brief Pain Inventory (BPI) are new tools developed to assess the pain in quantitative terms. The VAS has been validated (Porela-Tiihonen et al., 2013a) and used in previous studies to assess the effect of intracameral lidocaine on ocular pain (Tan et al., 2011; Porela-Tiihonen et al., 2013a; Hovanesian et al., 2015).

The BPI allows patients to rate the severity of the pain and grade how their pain interferes with common feelings and functions. Although BPI was initially developed to assess pain related to cancer (Porela-Tiihonen et al., 2013a), it has been shown to be an appropriate tool to measure pain elicited by a wide range of clinical conditions (Ehde et al., 2015).

To date, some studies have compared different types of cataract surgery anesthesia (Martin et al., 1998; Crandall et al., 1999; Oğurel et al., 2018). However, these trials investigated only off-label formulations (Crandall et al., 1999), compared various types of topical anesthesia (Crandall et al., 1999), used a single psychometric tool for pain measurement (Porela-Tiihonen et al., 2013b), considered additional postoperative analgesia (Donnenfeld et al., 2017), or had only a small sample size (Coelho et al., 2015). To the best of our knowledge, no study has compared the effect of Mydrane with other off-label intracameral anesthetic formulations.

The aim of the present study was to compare patient-reported pain intensity of two types of intracameral anesthetic solutions (the study drug or traditional 1% lidocaine/0.025% adrenaline formulation) for phacoemulsification and identify the factors affecting pain intensity using VAS and BPI in both groups. The null-hypotheses being tested is that eyes randomized to the study drug will have postoperative pain intensity the same or less than those randomized to traditional intracameral Lidocaine formulation.

## Materials and Methods

The study was conducted at the Department of General Ophthalmology of Medical University in Lublin, Poland. The study protocol conformed to the principles of the Declaration of Helsinki and was approved by the Local Research Ethics Committee (n° KE-0254/342/2018) and was registered on ClinicalTrials.gov (ID: NCT04166578).

A written informed consent form, for the processing of personal data, was obtained from all patients.

It was a prospective, one-center, single-blind, pilot, randomized study. Patients were randomly selected to the group receiving intracameral the study drug - manufactured solution of 0.02% tropicamide/0.31% phenylephrine/1% lidocaine (Mydrane™) (0.2 ml) or traditional intracameral formulation based on 1% lidocaine and 0.025% adrenaline (0.2 ml) during phacoemulsification.

Inclusion criteria were as follows: age above 18 years, best corrected visual acuity (BCVA) of 0.2 logMAR or worse, and agreement for taking part in the study.

Exclusion criteria were as follows: psychiatric diseases, epilepsy, ongoing treatment with hypnotics or psychotropic drugs (including opioids) within a week before admission, daily analgesic treatment, intake of additional rescue medications due to the pain after surgery, omitting postoperative visits. The patients who later needed additional medications for pain relief were excluded as it would be difficult to assess which medication is responsible for the achieved scores in psychometric measurements.

After inclusion in the study, the enrolled patients were randomly assigned, in a 1:1 ratio, to one of two groups related to the type of anesthesia, in a single-blind manner using computer-generated codes.

Diabetes mellitus was noted in 28.57% (n = 18) (9 in the study group and 9 in the reference group).

### Patient Preparation and Anesthesia

All patients received 1 drop of levofloxacin (Oftaquix™, Santen) at 60 min, 30 min, and 15 min before surgery. Topical anesthesia with 1 drop of 0.5% proparacaine (Alcaine™, Alcon) was given to all the patients at 30 min, 15 min, and 5 min before surgery. No general sedation was administered before surgery.

### Surgical Technique

After asepsis of the periocular skin with chlorhexidine 0.5% and a drop of povidone-iodine 5.0%, the eyelid speculum was carried out. The Constellation System (Alcon Laboratories, Inc., Forth-Worth, USA) was used with the standardized use of a microtip 30-gauge cannula. All surgeries (half to half) were performed by two experienced surgeons (RR and KN). The main incision was created on 3 planes of approximately 1.8 mm of extension with a triangular blade of 2.75 mm at the 10 o’clock position. In the Mydrane group, 0.2 ml of Mydrane™ was injected into the anterior chamber. In the reference group, a combination of lignocaine 1% lidocaine and 0.025% adrenaline (0.2 ml) was injected into the anterior chamber. Next, the anterior chamber was filled with methylcellulose 2.0%, and paracentesis in the surgical limbus was performed at the 2 o’clock position. A continuous curvilinear capsulorhexis approximately 5.5 mm in diameter, a hydrodelineation, and hydrodissection with balanced salt solution (BSS, Alcon Laboratories, Inc.) were performed. A stop-and-chop technique was performed. The cortex was aspirated with an irrigation/aspiration pen, and the IOL was implanted in the capsular bag using an injector and a cartridge. The incision was sealed by incisional edema with BSS. During the first 7 days after surgery, levofloxacin (Oftaquix) eye drops were applied every 4 h in combination with 0.1% dexamethasone (Dexafree) in a tapering schedule over the subsequent 20 days.

### Pain Measurement

**Figure 1:**
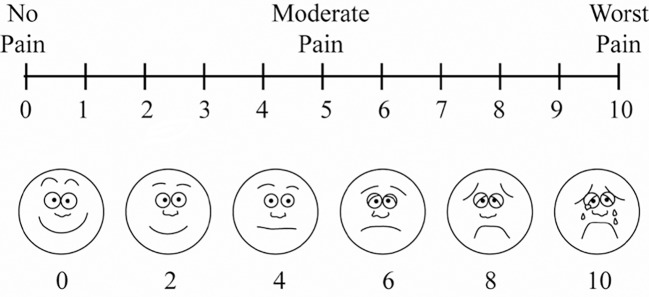
The Visual Analog Scale (VAS) for Pain.

The pain was assessed quantitatively 15 min before and after the end of surgery with VAS by an ophthalmologist other than the surgeon, and masked to the used anesthetic method. A unidimensional VAS 10 cm in length (equivalent to 10 degrees) was used, with its numbers (degrees) being visible only on the side of the examiner (**Figure 1**) (Scott, 1976). Before the measurement, the examiner explained to the patient that the 0 points represented no pain and that the 10 points represented the most intense pain he or she felt throughout the surgical procedure.

A third measurement was taken the next day after surgery using the BPI-short form, which is a widely used measurement tools for assessing clinical pain. It contains two domains that measure pain intensity (severity) and the impact of pain on functioning (interference). In the current study, BPI was used to evaluate the severity of pain and the impact of pain on daily function in the previous 24 h. The responses were given using an eleven-point numeric rating scale (NRS) scored 0–10, where 0 = best outcome/does not interfere/no pain/complete pain relief and 10 = worst outcome/completely interferes/most pain/no pain relief.

### Ophthalmological Examination

A full ophthalmological examination had been carried out before and 7 days after the surgery. BCVA, slit-lamp biomicroscopy and fundus examination were evaluated. The intraocular pressure was also measured. Coexisting eye diseases and post-operative complications were noted.

### Postoperative Period

The patients were not offered any oral or topical anesthetics until they filled out the BPI. None of the patients reported a need to use any analgetics after surgery.

### Statistical Analysis

The obtained results were subjected to statistical analysis. The values of the measurable parameters analyzed were presented using the mean value, median, quartiles, and standard deviation, and for the unmeasurable ones using the number and percentage. For measurable features, the normality of the distribution of the analyzed parameters was evaluated using the W Shapiro-Wilk test.

To compare the two groups, the Mann-Whitney test was used. The Spearman R test was used to assess the relationship between variables. For dependent variables, the Wilcoxon Pair Order test or the Character test was used. For unrelated qualitative features, the Chi2 homogeneity test was used to detect the existence of differences between the compared groups. The Chi2 test of independence was used to investigate the existence of dependencies between the examined features.

A *p* value ≤ 0.05 was considered statistically significant. All calculations were performed using STATISTICA 10 PL StatSoft.

## Results

**Figure 2:**
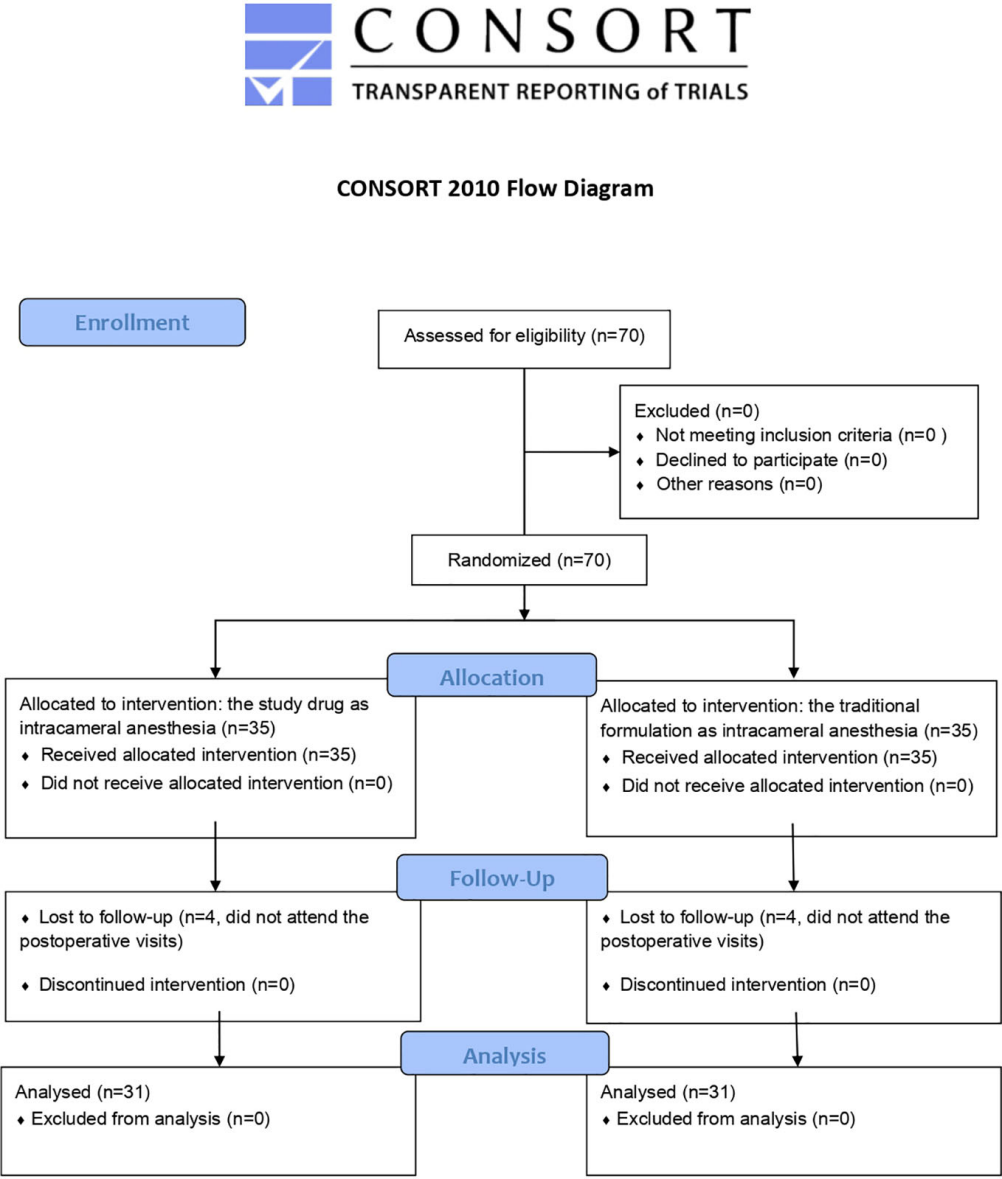
CONSORT 2010 Flow Diagram.

Initially, a total of 70 patients were scheduled to participate in the study. However, 8 patients did not attend postoperative visits and were excluded. As a result, only 62 patients were included in the analysis (**Figure 2**) (**Datasheet 1**).

The mean age of patients was 73.95 ± 7.82 years (Me = 73.00; range from 55-89 years). In the study group, the patients’ age was 75.20 ± 8.46, while in the control group - 72.45 ± 5.89. The ratio of women to men was 55.13% vs. 45.88% in the study drug group and 53.61% vs. 47.39% in the traditional formulation group. The average duration of surgery in the whole group was 11.53 ± 3.30 min. In the study group, the operation time was 11.61 min, while in the control group 11.44 min. The obtained results are presented in **Table 1**.

**Table 1:** 7The groups characteristics.

Variables		The study drug group		The traditional formulation group	
		N	%	n	%
Sex	M	15	46,88	15	48,39
	F	17	53,13	16	51,61
Age	Average ± SD (Me [min-Max])	75,20 ± 8,46 (Me=77,50; 55–89)		72,45 ± 5,89 (Me=73,00; 62–86)	
Surgery time	Average ± SD (Me [min-Max])	11,61± 3,35 (Me=10,00; 5–20)		11,44 ± 3,31 (Me=10,00; 5–20)	
Which surgery	First	25	78,13	26	83,87
	Second	7	21,88	5	16,13
Operating eye	Left	16	50,00	16	51,61
	Right	16	50,00	15	48,39
Comorbidities					
Myopia		2	6,24	7	22,58
AMD		7	22,58	6	19,35
ERM		1	3,13	1	3,13
Retinitis pigmentosa		1	3,13	0	0
PEX		1	3,13	1	3,23
Galucoma		0	0	1	3,23
No		19	59,37	15	48,38

### Pain Scores

At baseline before the surgery, 7 patients (11%)—5 in the study drug group and 2 in the traditional formulation group—reported ocular pain. The mean pain measured with VAS Pain was 0.34 in the study drug group and 0.09 in the traditional formulation group. There were no statistically significant differences between the two groups (*p* = 0.51). Moreover, the mean pain (± SD) measured with VAS Pain at the end of the surgery also revealed no statistically significant differences between the study drug and traditional formulation groups (1.15 ± 1.8 vs. 1.9 ± 2.7, respectively; *p* = 0.3).

The range of pain measured on the next day after surgery with BPI-short form was as followed:

1. There were no differences in the mean severity score between the study drug and the traditional formulation groups (*p* = 0.94).
2. There were no statistically significant differences between the two groups with respect to the influence of pain during the last 24 h on activity (*p* = 0.79), mood (*p* = 0.31), social contacts (*p* = 0.29), sleep (*p* = 0.5) and the joy of life (*p* = 0.39). We found only statistically significant differences in the ability to walk (*p* = 0.03) and everyday duties (*p* = 0.01).
3. Pain interference mean score of 7 domains (general activity, mood, walking ability, normal work, relations with other people, sleep, enjoyment of life) was significantly higher in the study drug group as compared to the traditional formulation group (1.41 vs. 0.69, respectively; *p* = 0.03) (**Table 2**).

**Table 2:** VAS and BPI values for Study Group (Mydrane) and Reference Group (solution of lignocaine 1% with adrenaline 0.025%).

	Study group	Reference group	p*
*Mean pain measured by VAS*	1.15	1.9	0.3
*Mean severity score measured by BPI*	1.29	1.16	0.9
*The strongest pain measured by BPI*	1.75	1.8	0.82
*Pain interference mean score by BPI*	1.41	0.69	0.03*

*A p value ≤ 0.05 was considered statistically significant.

### Factors Affecting Patients Pain Intensity

In addition to cataract, other ophthalmological conditions/diseases were observed in 13 (42%) patients in the study drug group and 17 (54%) patients in the traditional formulation group. These conditions were glaucoma (only in reference group, n = 1), diabetic retinopathy (the study drug group, n=3; the traditional formulation group, n = 7), retinitis pigmentosa (only in the study drug group, n = 1), age-related macular degeneration (the study drug group, n=7; the traditional formulation group, n=6), idiopathic epiretinal membrane (the study drug group, n=1; the traditional formulation group, n=1), pseudoexfoliation syndrome (the study drug group, n=1; the traditional formulation group, n=1), and high myopia (only in the study drug group, n=1). Moreover, there were no statistically significant differences between the two groups (*p* = 0.98).

#### Lateralization

Sixteen (52%) patients in the study drug group and 14 (45%) in the traditional formulation group underwent operation of the right eye. For 7 patients in the study drug and 5 patients in the traditional formulation group, the cataract surgery was performed on the first eye.

We did not find statistically significant differences between the two groups (*p* = 0.4) regarding postoperative complications. Eleven cases in the study drug group had corneal edema. In the traditional formulation group, 5 patients had corneal edema, 2 postoperative rise of intraocular pressure, 1 iris damage, and 1 hemorrhage into the anterior chamber.

There was no statistically significant influence of age, sex, lateralization, co-existing ophthalmological diseases, time of surgery, and post-operative complications on the experience of pain measured with VAS Pain during the surgery and on pain severity mean score and pain interference measured with BPI-short form (*p* = 0.2, 0.07, 0.07, 0.3, 0.1, respectively). The factors affecting pain during surgery and the time of the surgery influencing on pain in the traditional formulation and the study drug group are listed in **Tables 3**–**6**.

**Table 3:** Factors conditioning pain during cataract surgery in reference group.

Factors		Pain		No pain		Analysis
		n	%	N	%	
Sex	F	7	41,18	10	58,82	Chi^2^ = 0,21; *p* = 0.65
M	5	33,33	10	66,67		
Which surgery	First	4	57,14	3	42,86	Chi^2 =^ 1,48; *p* = 0.22
Second	8	32,00	17	68,00		
Comorbidities	No	7	36,84	12	63,16	Chi^2^ = 0,08; *p* = 0.78
Yes	5	38,46	8	61,54		
Complication	No	7	33,33	14	66,67	Chi^2^ = 0,45; *p* = 0.50
Yes	5	45,45	6	54,55		

**Table 4:** Factors conditioning pain during cataract surgery in Mydrane group.

Factors		Pain		No pain		Analysis
		n	%	N	%	
Sex	F	10	62,50	6	37,50	Chi^2^ = 1,57; *p* = 0.21
	M	6	40,00	9	60,00	
Which surgery	First	2	40,00	3	60,00	Chi^2^ = 0,32; *p* = 0.57
	Second	14	53,85	12	46,15	
Comorbidities	No	9	64,29	5	35,71	Chi^2^ = 1,64; *p* = 0.20
	Yes	7	41,18	10	58,82	
Complication	No	11	50,00	11	50,00	Chi^2^ = 0,08; *p* = 0.78
	Yes	5	55,56	4	44,44	

**Table 5:** Duration of surgery with the occurrence of pain during surgery in the study group.

Pain	Mediana	Standrad deviation	Inferior quartille	Mediana	Superior quartille
No	11.05	3.57	10.00	10.00	15.00
Yes	12.78	2.64	10.00	15.00	15.00

Statistical analysis: Z = −1,28; p = 0.20.

**Table 6:** Duration of surgery with the occurrence of pain during surgery in the Mydrane group.

Pain	Mediana	Standrad deviation	Inferior quartille	Mediana	Superior quartille
No	11.43	3.06	10.00	10.00	15.00
Yes	11.46	3.69	10.00	10.00	14.00

Statistical analysis: Z = 0,19; p = 0.85.

In the traditional formulation group, the patients who underwent second versus first cataract surgery showed a significant higher pain interference (*p* = 0.03) but similar mean severity pain score. Moreover, the mean severity pain score and pain interference did not differ in the study drug group when the order of the surgery was considered.

### Visual Acuity

BCVA was similar after the operation in both groups (p=0.66). Mean BCVA (± SD) after surgery was lower in the study drug group vs. the traditional formulation group (0.29 [± 0.66] logMAR vs. 0.36 [± 0.61] logMAR, respectively). When preoperative and postoperative BCVA values were compared, visual acuity improved significantly in both groups (*p* = 0.0008 vs. *p* = 0.0006).

There was a significant dependence between postoperative BCVA and co-existing diseases of the eye (*p* = 0.003) in the study drug group. A correlation between BCVA and postoperative complications in the traditional formulation group (*p* = 0.004) was noted. Postoperative BCVA did not correlate with pain intensity measured with VAS and BPI (the mean severity pain score, pain interference) in both groups.

## Discussion

To our knowledge, this is the first comparative study assessing the outcomes of two intracameral anesthesia in patients undergoing cataract surgery, except for the registration study (Labetoulle et al., 2016). In our study, we did not find any significant differences between the intracameral injection of the study drug Mydrane™ or the traditional combination of 1% lidocaine with 0.025% adrenaline regarding the intensity of pain referred during the surgery and its impact during the post-operative 24 h in terms of activity, mood, social contacts, sleep and the quality of life. However, there was a statistically significant difference when the impact of pain on the ability to walk and on everyday duties were evaluated, in favor of the reference group.

To estimate the pain intensity, we used two psychometrical tools: VAS Pain and BPI. The mean pain intensity measured with VAS Pain at the end of surgery was on the level 1.15 (SD ±1.8) in the study drug group and 1.90 (SD ± 2.7) in the traditional formulation group. These results are comparable with that of Tan et al.’s study (Tan et al., 2011). Also, Khezri and Merate (2013) used VAS to obtain pain and anxiety score during cataract surgery under topical anesthesia. They observed reduced anxiety scores in patients who received sublingual melatonin premedication. In their study, the median score in VAS measured at the recovery room was 1 with a range of 0 to 4 in the placebo group and 0 to 5 in the melatonin group. The pain intensity noted in our study was lower than in the study designed by Coelho et al. (2015). Authors examined the effect of cryoanalgesia during cataract surgery and found that there was no statistically significant difference with or without cryoanelgesia during the procedure (Coelho et al., 2015). The VAS Pain results showed that the range of pain during surgery was 2.13 ± 0.36 (SD) in the group with cryoanalgesia and 2.6 ± 0.37 (SD) in reference group. Moreover, in a study evaluating analgesic effectiveness of 0.1% nepafenac during cataract surgery, the pain intensity was higher than our scores (Oğurel et al., 2018). Mean VAS Pain score (± SD) was 2.15 (± 1.23) in the treatment group and 4.15 (± 1.13) in the placebo group (*p* = 0.024). Porela-Tiihonen et al. (2013b) evaluated postoperative pain in 196 patients who underwent elective first eye cataract extraction surgery using BPI. Postoperative pain was relatively common during the first hours after surgery as reported by 67 (34%) patients. Most of these patients reported a significant pain, with a score of ≥ 4 on a pain scale of 0–10. In our study, 12 (37%) patients in Mydrane™ group and 15 (46%) patients in control group reported pain during phacoemulsification, and the mean measured with VAS Pain was lower than in the evoked study. Only 4 (12%) patients in the study drug group and 5 (15%) patients in the traditional formulation group scored 4 points or more as pain during cataract surgery. The rest of our patients reported pain with a score lower than 4 or without any pain. The mean pain severity score (± SD) was 1.29 (± 1.57) in the study drug group and 1.16 (± 1.42) in the traditional formulation group. Moreover, Tan et al. (2011) did not calculate the mean pain severity score, which is recognized as a valuable factor, when describing pain intensity. In comparison, Crandall et al. (1999) showed that the mean intraoperative pain score measured with VAS was 0.86 in the lidocaine group and 1.2 in the placebo group. These results are lower than in our study, but we believe that intravenous sedation executed in some patients may have influenced the score. In a randomized clinical trial including 223 patients, Donnenfeld et al. evaluated Omidria (phenylephrine and ketorolac injection 1.0%/0.3%) compared with a BSS (vehicle), ketorolac, and phenylephrine on early postoperative ocular pain (Donnenfeld et al., 2017). The ocular pain within 12 h after surgery measured using VAS was significantly lower in Omiridia group. Moreover, the authors did not emphasize the exact range of pain score.

Previous studies have also evaluated factors affecting pain intensity during cataract surgery (Rifkin and Schaal, 2012; Bjørnnes et al., 2016). In our study, patients who had previously undergone cataract surgery to the fellow eye were more likely to experience pain compared to those undergoing it for the first time. This finding has been previously reported both in cataract (Hovanesian et al., 2015) and vitreoretinal surgery (Porela-Tiihonen et al., 2013b). Conversely, Ogurel et al. did not find statistically significant difference in mean VAS pain score when first and second eyes were compared (Oğurel et al., 2018).

We suspected that age might have affected the pain during surgery. It has been shown that aging may impact pain perception and expression (Omar et al., 2012; Tighe et al., 2015; Lesin et al., 2016). Unpleasant and severe ocular irritation symptoms may not be reported as pain related to the surgery by elderly patients, as observed in the present study. Furthermore, each individual has a subjective definition of pain, and may use euphemisms when describing pain conditions in special emotional experiences. Thus, pain assessment is a challenging task in clinical work. According to the definition of the International Association for the Study of Pain, pain is “an unpleasant sensory and emotional experience associated with actual or potential tissue damage, or described in terms of such damage” (Bonica, 1979) it may be difficult to distinguish them from postoperative pain. Additionally, pain reveals a cognitive perception, ie. patients give different meanings to the postoperative complaints they have. Nevertheless, we did not find any influence of age on pain, as already reported by Tan et al. in a study on the effect of supplemental intracameral lidocaine 1% during phacoemulsification under topical anaesthesia (Tan et al., 2011).

Our results also showed no correlation between gender and pain, as previously shown by Coelho et al. (2015). Contrarily, some authors described the female gender as a predictor of increased postoperative pain intensity (Lesin et al., 2015). However, the review research has some limitations and included different types of ocular surgeries.

Also, lateralization did not influence pain during phacoemulsification. This finding is similar to the results shown by Tan et al. (2011) who found that the operated eye (right or left) did not affect the pain experienced. To our knowledge, there are no studies describing the influence of lateralization on pain during cataract surgery.

In our study, we did not find any correlation between other ophthalmological conditions, postoperative complications, and pain. Some authors tried to determine factors affecting patients’ pain intensity during “in office” intravitreal injection procedure but did not observe correlation between ophthalmological diagnosis and pain intensity (Rifkin and Schaal, 2012). Similar outcomes were found for other ophthalmic surgical procedures (Lesin et al., 2016). The association of postoperative complications with pain intensity has not been examined yet.

In our study, visual acuity improved after cataract surgery, but it did not affect pain severity and pain interference. In a previous study about intravitreal injection, visual acuity improvement did not decrease pain intensity before the procedure (Rifkin and Schaal, 2012).

According to the mechanism of pain development during cataract surgery, there are two possibilities. Firstly, the surgical manipulation triggers the arachidonic acid cascade, which is responsible for the production of inflammatory mediators such as prostaglandins (Ahuja et al., 2008; Kohnen, 2015). Clinical signs of increased prostaglandins production, except pain, include inflammation, which is classically treated with steroids and nonsteroidal anti-inflammatory drugs (NSAIDs) (Kohnen, 2015). Numaga et al. found that 0.1% nepafenac seems to be a safe and efficacious ophthalmic suspension for postoperative inflammation and eye pain following cataract surgery or other ophthalmic procedures (Numaga et al., 2012). On the other hand, Ogurel et al. evaluated analgesic effectiveness and patient satisfaction of 0.1% nepafenac in patients who underwent bilateral cataract surgery with VAS and VPS (Oğurel et al., 2018). They found that 0.1% nepafenac seems to reduce pain in case of routine clear corneal phacoemulsification with topical anesthesia and increase intraoperative patient comfort if used preoperatively. Mean VAS pain score was 2.15 ± 1.23 in the group treated with 0.1% nepafenac and 4.15 ± 1.13 in the group without NASID (Porela-Tiihonen et al., 2013b). In our study, the mean VAS pain score was lower possibly due to the different mechanism of action of intracameral agents.

Second, the stimulation of ciliary nerves during manipulation of the iris and stretching of the ciliary and zonular tissues can produce discomfort. An injection of 1% lidocaine through the side port incision provides sensory blockage of the iris and ciliary body (trigeminal nerve endings) and, thereby, relieves discomfort experienced during IOL placement (Martin et al., 1998).

According to our results, the study drug Mydrane™ and the traditional anesthetic formulation seems to have similar efficacy in attenuating pain. Other studies indicated Mydrane™ to be effective also as an intraoperative mydriatic (Labetoulle et al., 2016; Schulz et al., 2018). For these considerations, Davey et al. shown that Mydrane™ is a cost effective and safe procedure in United Kingdom (Davey et al., 2018). On the contrary, the cost of off-label anesthetic formulation has not been investigated and remain unclear.

In summary, our study shows that the study drug Mydrane™, seems to be as effective as off-label anesthetic formulation, in reducing the pain experienced during cataract surgery under topical anesthesia, improving patients’ cooperation and satisfaction. Although several combinations of intracameral agents are being used during cataract replacement procedures, the only EMA-approved product for this use is Mydrane™ formulation. Further randomized clinical trials with a larger sample size are required to better investigate the effect of Mydrane™ formulation during complicated cataract surgery and other surgical ocular procedures.

## Data Availability Statement

All datasets generated for this study are included in the article/**Supplementary Material**.

## Ethics Statement

The studies involving human participants were reviewed and approved by Local Research Ethics Committee, Department of General Ophthalmology, Medical University of Lublin, Poland (KE-0254/342/2018). The patients/participants provided their written informed consent to participate in this study.

## Author Contributions

MT and RR: conceptualization, design of the study and project administration as Principals Investigators. MT and DN wrote the first draft of the manuscript. MR, TA, KN, CB and CC: revision and validation of the manuscript. AB and PM: data curation and statistical analysis. All authors gave final approval of the submitted version.

## Conflict of Interest

The authors declare that the research was conducted in the absence of any commercial or financial relationships that could be construed as a potential conflict of interest.

## Footnotes

1 http://apps.who.int/iris/bitstream/10665/93142/1/EML_18_eng.pdf?ua=1

2 https://www.nice.org.uk/guidance/ng77/chapter/Recommendations#surgical-timing-and-technique
